# Dynamical Mechanisms for Gene Regulation Mediated by Two Noncoding RNAs in Long-Term Memory Formation

**DOI:** 10.1155/2021/6668389

**Published:** 2021-03-25

**Authors:** Lijie Hao, Zhuoqin Yang

**Affiliations:** ^1^School of Mathematics Science, Tianjin Normal University, Tianjin 300387, China; ^2^School of Mathematics and Systems Science and LMIB, Beihang University, Beijing 100191, China

## Abstract

Noncoding RNAs such as miRNAs and piRNAs have long-lasting effects on the regulation of gene expression involved in long-term synaptic changes. To characterize gene regulation mediated by small noncoding RNAs associated with long-term memory in *Aplysia*, we consider two noncoding RNAs stimulated by 5-HT into a gene regulatory network motif model, including miR-124 that binds to and inhibits the mRNA of CREB1 and piR-F that facilitates serotonin-dependent DNA methylation to lead to repression of CREB2. Codimension-1 and -2 bifurcation analyses of 5-HT regulating both miR-124 and piR-F and a negative feedback strength for oscillation reveal rich dynamical properties of bistability and oscillations robust to variations in all other parameters. More importantly, we verify three stimulus protocols of 5-HT in experiments by our model and find that application of five pulses of 5-HT leads to a transient decrease of miR-124 but increase of piR-F concentrations, which matters sustained high level of CREB1 concentration associated with long-term memory. Furthermore, we perform bifurcation analyses for the concentrations of miR-124 and piR-F as two parameters to explore dynamical mechanisms underlying the epigenetic regulation in long-term memory formation. This study provides insights into revealing regulatory roles of epigenetic changes in gene expression involving noncoding RNAs associated with synaptic plasticity.

## 1. Introduction

Learning and memory are two of the most critical brain functions for acquiring new knowledge from experience and retaining that knowledge over time [1–3]. Sensitization of gill-withdrawal reflexes of the marine mollusc *Aplysia*, a simple form of long-term memory (LTM) that is possibly connected with serotonin (5-HT)-induced long-term facilitation (LTF) of sensorimotor neuron synapses, has been extensively studied for cellular and molecular mechanisms of LTM [4, 5]. In *Aplysia*, cyclic AMP- (cAMP-) response element-binding proteins (CREBs) as transcription factors (TFs) are crucial for the regulation of the gene expression required for neuronal plasticity and formation of LTM [6]. CREB1 functions as a transcriptional activator necessary for induction of LTF, while CREB2 is a transcriptional repressor that poses inhibitory constraints on the induction and formation of LTM.

Epigenetic mechanisms, which change gene expression but not the underlying DNA, are widely known to be involved in the formation and long-term storage of cellular information in response to transient environmental stimuli [7–9]. There are two main types of epigenetic modification: DNA methylation and histone modification [10–12]. A large body of experimental evidence suggests that small regulatory noncoding RNAs can cause long-lasting changes in cellular phenotypes during development, through their involvement in both autoregulatory feedback loops [13, 14] and the transcriptional and epigenetic regulation of gene expression [15, 16].

There are two classes of small RNAs regulated by neural activity in *Aplysia*: microRNAs (miRNAs) and Piwi-interacting RNAs (piRNAs), and their expression in turn regulates transcriptional and posttranscriptional mechanisms [17]. In *Aplysia*, the most abundant miRNA specific to the brain is miR-124, which binds to and inhibits the mRNA of CREB1 in the sensory neuron [18]. The brain of *Aplysia* also contains another class of noncoding RNA molecules, piRNA, such as piR-F, which leads to the DNA methylation as an epigenetic modification and the repression of the promoter of CREB2 [19]. Serotonin regulates piRNA molecules through a rise in piR-F silences CREB2 and miRNA molecules when a drop in miR-124 activates CREB1, which both establish stable, long-term changes in the sensory neurons that store memory [7]. These experimental findings reveal epigenetic mechanisms underlying regulations of the gene expression in long-term memory storage.

Recently, computational studies have identified an abundance of motifs involving noncoding RNAs and TFs [20–26]. For example, Zhou et al. [22] propose two particular network motifs: the miRNA-mediated double negative feedback loop in which a TF suppresses a miRNA, and the TF itself is negatively regulated by the miRNA, and the miRNA-mediated single negative feedback loop in which a TF activates a miRNA, and the TF itself is negatively regulated by the miRNA. Nitzan et al. [24] provide a theoretical and numerical study of coherent mixed feedback loops of two genes, in which a TF and a small noncoding RNA mutually regulate each other's expression. Hao et al. establish a network model of the regulation between CREB1 and miR-124 stimulated by 5-HT, which is associated with long-term memory formation in *Aplysia* [26]. However, these mathematical models did not involve piRNAs, which possibly play a role in the epigenetic regulation in gene expression through DNA methylation.

In this paper, we construct a mathematical model characterizing epigenetic regulation of gene expression by two noncoding RNAs related to LTM in *Aplysia*, in which miR-124 inhibits the mRNA of CREB1 and piR-F facilitates serotonin-dependent DNA methylation of CREB2 and silences it. Codimension-2 bifurcation analysis in the model indicates that the system displays complex dynamics, including bistability and oscillation, which are robust to variations in parameters. In addition, the system exhibits diverse codimension-1 bifurcations such as saddle-node bifurcation, saddle-node invariant circle bifurcation, Hopf bifurcation, fold limit cycle bifurcation, and homoclinic bifurcation. We further verify the model by four stimulus protocols of 5-HT used to simulate experiments of the induction of long-term memory in *Aplysia*. Finally, we consider the concentrations of miR-124 and piR-F as parameters and perform bifurcation analyses to reveal dynamical mechanisms underlying the epigenetic regulation in long-term memory formation.

## 2. Model

**Figure 1:**
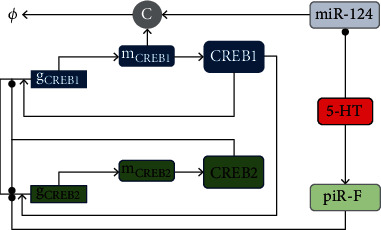
Illustration of the model. The noncoding RNA piR-F is activated while miR-124 is repressed by 5-HT. CREB1 activates the CREB2 gene and its own gene transcription, while CREB2 represses CREB1 gene and its own gene transcription. CREB1 mRNA (m_CREB1_) and miR-124 are combined into a complex (C). Arrows and black dots denote activation and suppression, respectively.

We establish a regulatory motif of two transcription factors (CREB1 and CREB2) and two noncoding RNAs (miR-124 and piR-F) stimulated by 5-HT, where CREB1 and CREB2 are negatively regulated by miR-124 and piR-F, respectively (see Figure 1). According to the epigenetic mechanism of long-term memory in *Aplysia* [7], miR-124 may bind CREB1 mRNA to inhibit its translation, and piRNA facilitates serotonin-dependent methylation and thus represses the promotor of CREB2. Interval applications of five pulses of 5-HT to the sensory neurons can reduce the expression of several miRNAs including miR-124 [18] but upregulate piR-F level [19]. The activator CREB1 would activate but the repressor CREB2 would repress the expression of CREB1 gene (g_CREB1_) and CREB2 gene (g_CREB2_), respectively.

The levels of CREB1 mRNA, CREB2 mRNA, miR-124 mRNA, and piR-F mRNA in the cell are denoted as mCREB1, mCREB2, miR-124, and piR-F, respectively. We define a function of 5-HT inhibition on miR-124 through *λ* and a function of 5-HT promotion on piR-F through *γ*. CREB1 mRNA and miR-124 are combined into a complex with a rate of *δ*, and then, the mCREB1-miR124 complex is presumed to be degraded rather than dissociated into its miR-124 and mCREB1 components. piR-F that leads to the methylation of the promoter of CREB2 and inhibits CREB2 gene expression is assumed to be described as *α*/(*β* + [piR‐F]). CREBs in the transcription of the CREB1 and CREB2 genes can be illustrated as (*V*_1_[CREB1]^2^)/([CREB1]^2^ + *K*_1_^2^) · ([CREB2]^2^)/([CREB2]^2^ + *K*_2_^2^) and (*V*_2_[CREB1]^2^)/([CREB1]^2^ + *K*_1_^2^) · ([CREB2]^2^)/([CREB2]^2^ + *K*_2_^2^), respectively, where *V*_1_ and *V*_2_ are the feedback strengths between CREB1 and CREB2, and *K*_1_ and *K*_2_ are two dissociation constants of two complexes of CREB1 and CREB2 from the promoter regions of the CREB genes. The Hill coefficients of 2 for [CREB1] and [CREB2] represent the requirement for two CREB1 or CREB2 monomers to form homodimers [6, 27]. Basic transcription rates of g_CREB1_ and g_CREB2_ and translation rates of m_CREB1_ and m_CREB2_ are denoted by *g*_*m*1_, *g*_*m*2_, *k*_*p*1_, and *k*_*p*2_, respectively. The degradation rates of miR-124, piR-F, m_CREB1_, m_CREB2_, CREB1, and CREB2 are defined by *d*_*i*_, *d*_*p*_, *d*_*m*1_, *d*_*m*2_, *d*_*p*1_, and *d*_*p*2_, respectively.

We depict the gene regulatory network model by rate Equations (1)–(6). Suitable values of all important coefficients within their biological range of experiments are listed in Table 1. The choice of parameter values is mostly from previous models of long-term memory in *Aplysia* [6, 27] or is made to fit the experimental data. All numerical simulations are executed through the Runge-Kutta method [28] as well as bifurcation analyses are performed with XPPAUT.

(1) $$\begin{array}{c} \frac{\text{d}\left[\text{miR}‐124\right]}{\text{d}t}=g_{i}-\lambda \left[5‐\text{HT}\right]\left[\text{miR}‐124\right]-\delta \left[\text{miR}‐124\right]\left[{\text{m}}_{\text{CREB}1}\right]-d_{i}\left[\text{miR}‐124\right], \end{array}$$

(2) $$\begin{array}{c} \frac{\text{d}\left[\text{piR}‐\text{F}\right]}{\text{d}t}=g_{p}+\gamma \left[5‐\text{HT}\right]-d_{p}\left[\text{piR}‐\text{F}\right], \end{array}$$

(3) $$\begin{array}{c} \frac{\text{d}\left[{\text{m}}_{\text{CREB}1}\right]}{\text{d}t}=g_{m1}+\frac{V_{1}{\left[\text{CREB}1\right]}^{2}}{{\left[\text{CREB}1\right]}^{2}+K_{1}^{2}}\cdot \frac{{\left[\text{CREB}2\right]}^{2}}{{\left[\text{CREB}2\right]}^{2}+K_{2}^{2}}-\delta \left[\text{miR}‐124\right]\left[{\text{m}}_{\text{CREB}1}\right]-d_{m1}\left[{\text{m}}_{\text{CREB}1}\right], \end{array}$$

(4) $$\begin{array}{c} \frac{\text{d}\left[\text{CREB}1\right]}{\text{d}t}=k_{p1}\left[{\text{m}}_{\text{CREB}1}\right]-d_{p1}\left[\text{CREB}1\right], \end{array}$$

(5) $$\begin{array}{c} \frac{\text{d}\left[{\text{m}}_{\text{CREB}2}\right]}{\text{d}t}=g_{m2}+\frac{V_{2}{\left[\text{CREB}1\right]}^{2}}{{\left[\text{CREB}1\right]}^{2}+K_{1}^{2}}\cdot \frac{{\left[\text{CREB}2\right]}^{2}}{{\left[\text{CREB}2\right]}^{2}+K_{2}^{2}}\cdot \frac{\alpha }{\beta +\left[\text{piR}‐\text{F}\right]}-d_{m2}\left[{\text{m}}_{\text{CREB}2}\right], \end{array}$$

(6) $$\begin{array}{c} \frac{\text{d}\left[\text{CREB}2\right]}{\text{d}t}=k_{p2}\left[{\text{m}}_{\text{CREB}2}\right]-d_{p2}\left[\text{CREB}2\right], \end{array}$$

## 3. Results

### 3.1. Bifurcation Analysis with respect to the Stimulus 5-HT and the Negative Feedback Strength

Negative feedback has the potential to evoke limit-cycle oscillations which are crucial for explaining physiological rhythmicity in biochemical systems. In our model, the activator CREB1 activates expression of CREB2 gene but the repressor CREB2 in turn represses expression of CREB1 gene, which are together able to close a negative feedback loop with the negative feedback strength *V*_2_. Also, the negative feedback strength *V*_2_ represents the rate of CREB2 gene transcription regulated by the noncoding RNA piR-F considered significantly in the model. Here, focusing on the stimulus strength [5-HT] under the physiologically relevance of negative feedback strength *V*_2_, we explore diverse dynamics such as monostable, bistable, and oscillatory behaviors in the ([5‐HT], *V*_2_) parameter plane through codimension-2 bifurcation analysis.

**Figure 2:**
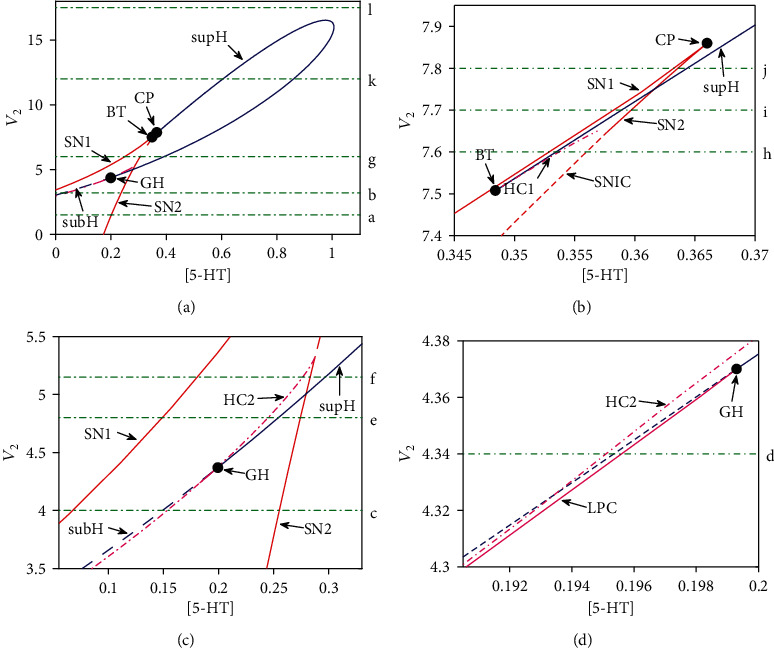
Bifurcation diagram in the ([5‐HT], *V*_2_) parameter plane. The red solid line, red dashed line, blue solid line, blue dashed line, magenta solid line, and magenta dash-dotted line depict the saddle-node bifurcation points (SN), saddle-node invariant circle bifurcation points (SNIC), supercritical Hopf bifurcation points (supH), subcritical Hopf bifurcation points (subH), fold limit cycle bifurcation points (LPC), and homoclinic bifurcation points (HC)), respectively. Three codimension-2 bifurcation points exist: a cusp point (CP), a Bogdanov-Takens bifurcation point (BT), and a generalized Hopf bifurcation point (GH). (b–d) are the enlargement of (a).

Two-parameter bifurcation diagram in the ([5‐HT], *V*_2_) plane is constructed by continuation of the loci of six different types of codimension-1 bifurcation points, namely, saddle-node bifurcation points (SN, red solid line), saddle-node invariant circle bifurcation points (SNIC, red dashed line), supercritical Hopf bifurcation points (supH, blue solid line), subcritical Hopf bifurcation points (subH, blue dashed line), fold limit cycle bifurcation points (LPC, magenta solid line), and homoclinic bifurcation points (HC, magenta dash-dotted line) (as shown in Figure 2). The SN1 and SN2 bifurcation curves coalesce at a codimension-2 cusp point (CP), whereas the SN1 bifurcation curve meet with the supH bifurcation curve at a codimension-2 Bogdanov-Takens bifurcation point (BT). The codimension-2 generalized Hopf bifurcation point (GH) corresponds to the meeting point of supH bifurcation curve and subH bifurcation curve at which the LPC bifurcation curve (magenta solid) occurs.

**Figure 3:**
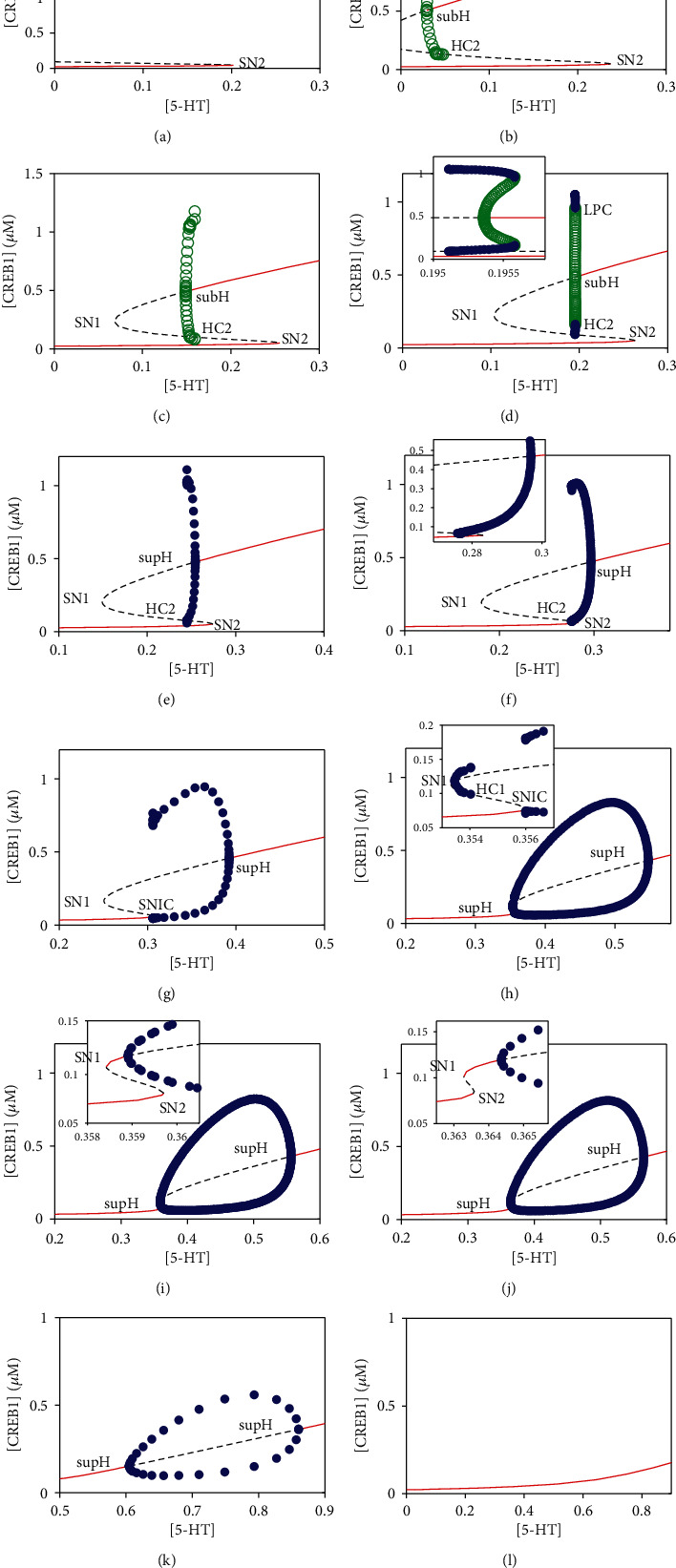
Bifurcation diagrams of [CREB1] versus [5-HT] at twelve values of *V*_2_. Stable and unstable steady states are represented by red solid lines and black dashed lines, respectively. For the stable and unstable limit cycles, the maximum and minimum values of [CREB1] are denoted by blue solid circles and green open circles, respectively. (a–l) correspond to *V*_2_ = 1.5, 3.2, 4, 4.34, 4.8, 5.15, 6, 7.6, 7.7, 7.8, 12, and 17.5, respectively, as marked in Figure 2.

To obtain a clear insight into the codimension-two bifurcation diagram in Figure 2, we consider a codimension-one bifurcation of the concentration of CREB1 with respect to the concentration of the stimulus 5-HT. The bifurcation diagram is illustrated in Figure 3 in which panels (a)–(l) correspond to *V*_2_ = 1.5, 3.2, 4, 4.34, 4.8, 5.15, 6, 7.6, 7.7, 7.8, 12, and 17.5, respectively, as marked in Figure 2. Diagrams for each *V*_2_ value are detailed below.

For a small value of *V*_2_ as 1.5, the system changes from bistability to monostability with the increasing [5-HT] via a saddle-node bifurcation (SN2) on the SN2 bifurcation curve in Figure 2 (see Figure 3(a)). As *V*_2_ is increased to 3.2 and even 4, an unstable limit cycle generated by a subcritical Hopf bifurcation (subH) on the upper branch grows gradually and then collides with a saddle at a homoclinic bifurcation (HC2) point on the middle branch in Figures 3(b) and 3(c). As *V*_2_ is fixed at 4.34 (see Figure 3(d)), a stable limit cycle and an unstable limit cycle appear pairwise around the high stable steady state via a fold limit cycle bifurcation (LPC). Furthermore, the unstable limit cycle shrinks as well as the stable steady states loses stability through a subcritical Hopf bifurcation (subH) with decreasing [5-HT]. When *V*_2_ is increased to 4.8 and 5.15, a stable limit cycle arises via a supercritical Hopf bifurcation (supH), which grows gradually and then collides with a saddle at HC2 point (see Figures 3(e) and 3(f)). Only difference of them is that bistability exists with a high and a low stable steady states and a saddle between the supH point and the SN2 point in Figure 3(e). However, different from that in Figures 3(e) and 3(f), the destiny of the stable limit cycle generated at the supH point as *V*_2_ = 6 in Figure 3(g) is that it vanishes via a saddle-node invariant circle bifurcation (SNIC) when the stable limit cycle meets with the saddle-node point. When *V*_2_ is increased to 7.6 (see Figure 3(h)), stable limit cycles can emerge through two supercritical Hopf bifurcations (supH) on the upper branch, which disappear via homoclinic bifurcation (HC1) and SNIC bifurcation, respectively. However, at *V*_2_ = 7.7, 7.8, and 12 (see Figures 3(i)–3(k)), the stable limit cycle generated by the supH bifurcation grows gradually with increasing [5-HT] and then shrinks and eventually vanishes at another supH bifurcation point. The differences of these three bifurcation diagrams are that two saddle-node bifurcations (SN1 and SN2) exist in Figures 3(i) and 3(j), where the left supH point locates between the SN1point and SN2 point in Figure 3(i). As *V*_2_ is increased to 17.5, the system keeps monostable in Figure 3(l).

The above analyses show that our model possesses abundant dynamical properties such as monostability, bistability, and oscillations, which themselves can make diverse transitions via complicated bifurcation mechanisms as shown in the codim-1 and -2 bifurcation diagrams.

### 3.2. Bistability and Oscillations in the Model Are Robust to Variations in Parameters

**Figure 4:**
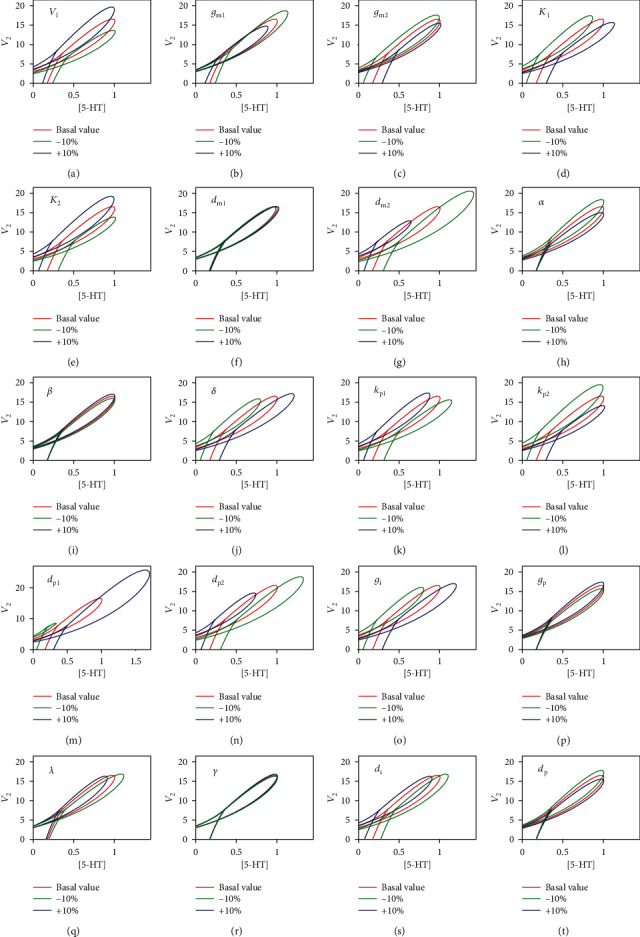
Impact of changing each parameter with ±10% on the two-parameter bifurcation diagram in the ([5‐HT], *V*_2_) plane. Red lines depict the loci of the saddle-node bifurcation points and Hopf bifurcation points with basal values, and green and blue lines correspond to parameter values scaled down and up 10 percent, respectively.

In Figure 2, saddle-node bifurcation and Hopf bifurcation for the parameter [5-HT] and the negative feedback strength *V*_2_ produce bistable and oscillatory dynamical behaviors, respectively, which are always important in the system dynamics with physiological significance. Therefore, it is necessary to discuss if the regions of bistability and oscillations in the parameters plane ([5‐HT], *V*_2_) are robust to variations in other parameters. One way to investigate the robustness of system dynamics is to vary the values of the other parameters in the system and observe changes in size and location of the regions where particular dynamics exist [6].  Figure 4 displays the two-parameter bifurcation curves after changing each parameter value with ±10% in the model in the ([5‐HT], *V*_2_) plane. Only the loci of the saddle node bifurcation points and the Hopf bifurcation points (subH and supH) are clearly displayed; however, both the fold limit cycle bifurcations (LPC) and the homoclinic bifurcations (HC) are too close to the Hopf bifurcation points to be hardly distinguished in the parameter planes ([5‐HT], *V*_2_). As shown in Figure 4, varying the values of the parameters makes the regions of bistability and oscillations change a little in both the size and the location, since it only moves the loci of the saddle-node points and the Hopf points. Indeed, the regions of bistability and oscillations are robust to variations of all the parameters.

### 3.3. Validation of the Model via Four Experimental Protocols under 5-HT Stimulation

In experiments, five pulses of treatment with 5-HT induce long-term facilitation (LTF) of synapses between sensory neurons and motor neurons in *Aplysia*, which correlates with long-term memory (LTM) formation, whereas one or three pulses do not [4, 29]. Empirically, sustained high level of CREB1 is often associated with memory storage [30], because the transcription factor CREB1 plays essential roles in the maintenance of LTM by activating a set of downstream genes to promote synaptic facilitation. Moreover, with exposure to five spaced pulses of 5-HT, the level of miR-124 begins to decrease, then slowly reaccumulates, and finally returns to baseline for a long time [18]. In contrast, the piR-F level with exposure to 5-HT is firstly upregulated and then drops back to baseline after stimulation [19].

**Figure 5:**
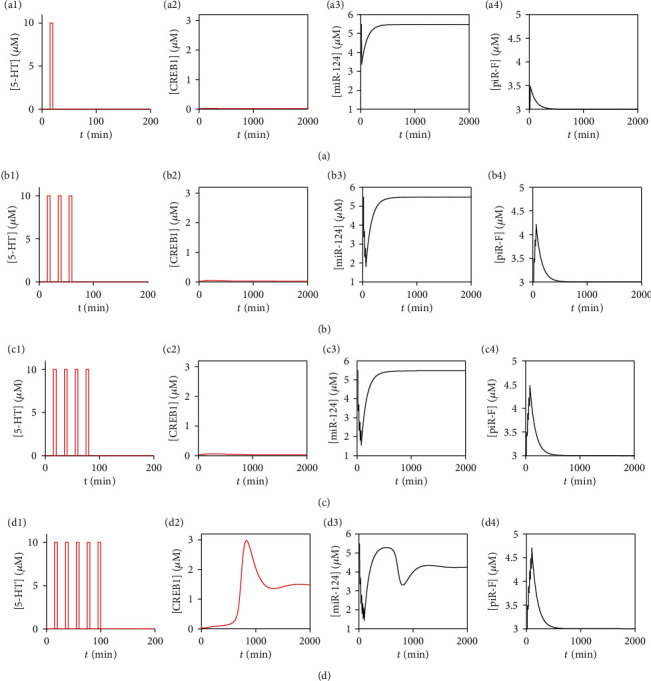
Time courses of [CREB1] (the second column), [miR-124] (the third column), and [piR-F] (the fourth column) under different stimulus protocols (the first column): one pulse (a), three pulses (b), four pulses (c), and five pulses (d) of 10*μ*M stimulus for 5 min and the interpulse interval (from the end of one pulse to the onset of the next) of 15 min. Other parameter values are given in Table 1.

Therefore, we focus on the dynamics of CREB1 as well as in four stimulus protocols for one (a), three (b), four (c), and five (d) short pulses applied in our model as presented in Figure 5. Application of 5-HT leads to a transient decrease in [miR-124] but increase in [piR-F], which both return to their original levels after stimulation. It is worthy to notice that the five pulses of 5-HT in Figure 5(d) make [miR-124] lower but [piR-F] higher than the pulses depicted in Figures 5(a)–5(c). In fact, only five short pulses can induce sustained high level of [CREB1] (see Figure 5(d2)), while one, three, or four short pulses fail to do it (see Figures 5(a2), 5(b2), and 5(c2)). These simulation results are qualitatively in accordance with experimental findings.

### 3.4. Dynamical Mechanisms Underlying the Epigenetic Regulation in Long-Term Memory Formation

**Figure 6:**
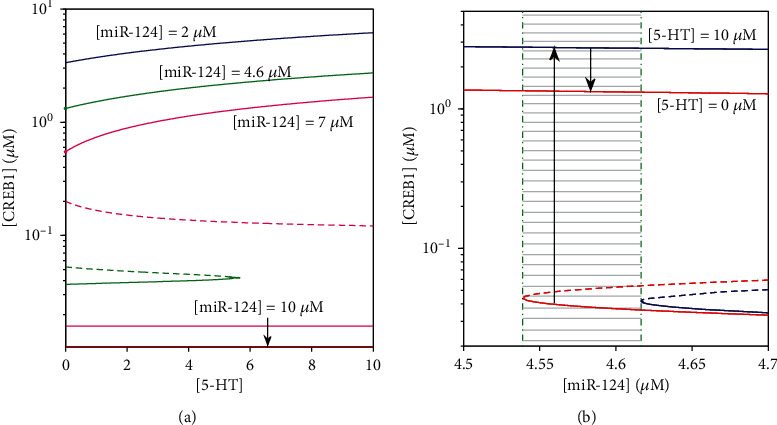
Bifurcation diagrams of [CREB1] versus [5-HT] for different [miR-124] (a) and [CREB1] versus [miR-124] at [5‐HT] = 0*μ*M (red lines) and [5‐HT] = 10*μ*M (blue lines) (b). Stable and unstable steady states are represented by solid lines and dashed lines, respectively.

**Figure 7:**
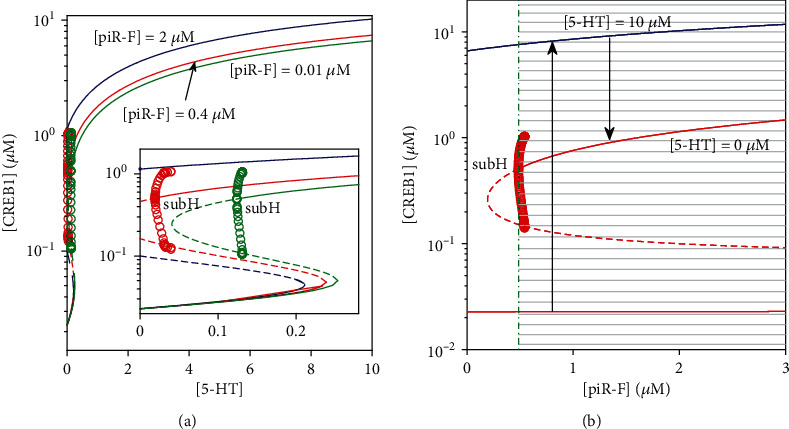
Bifurcation diagrams of [CREB1] versus [5-HT] for different [piR-F] (a) and [CREB1] versus [piR-F] at [5‐HT] = 0*μ*M (red lines) and [5‐HT] = 10*μ*M (blue lines) (b). Stable and unstable steady states are represented by solid lines and dashed lines, respectively. For the unstable limit cycles, the maximum and minimum values of [CREB1] are denoted by open circles.

To explore individual ability of miR-124 and piR-F to regulate the CREB1 level stimulated by 5-HT, we provide bifurcation analyses of [CREB1] versus [5-HT] at different values of [miR-124] (see Figure 6) and of [piR-F] (see Figure 7) to find appropriate ranges of [miR-124] and [piR-F] that allow the system to switch between two steady states. Generally, the existence of an irreversible bistable switch, that is, the system is bistable at basal level ([5‐HT] = 0*μ*M) but monostable with a high steady state at stimulation level ([5‐HT] = 10*μ*M). Therefore, the stimulus with enough duration makes the system transit from the low steady state to the high one and then keep high after stimulation. We will establish appropriate ranges of miR-124 and piR-F for state switching according to these dynamical features.

When [miR-124] is small as 2*μ*M (blue), the system is always monostable with a high steady state. As [miR-124] is increased to 4.6*μ*M (green), there exists an irreversible switch due to only one saddle-node bifurcation point in the physiological range of the parameter [5-HT]. Therefore, [CREB1] transits from low to high through the saddle-node bifurcation point as [5-HT] increases and still remains high even when [5-HT] decreases to the basal level afterwards. For [miR‐124] = 7*μ*M (pink), neither reversible switch nor irreversible switch exists since [5-HT] is unable to trigger a transition between the low and the high stable steady states with bistability. When [miR-124] is at a very high level as 10*μ*M (red), the system is invariably monostable with a low steady state. Furthermore, Figure 6(b) shows the range of [miR-124] between two dash-dotted line that enables the system to transit from low to high under 5-HT stimulation (see shaded part). The system with bistablility stays in the low-level steady state before stimulation ([5‐HT] = 0*μ*M), and then, it reaches the only high-level steady state at the stimulation [5‐HT] = 10*μ*M and persists high even after stimulation.

Unexpectedly, as [piR-F] is so small as 0.01*μ*M (green) or 0.4*μ*M (red), a subcritical Hopf bifurcation accompanied with only unstable limit cycle appears upper branch of the S-shaped bifurcation curve as shown in Figure 7(a). Thus, a reversible switch exists since the system is bistable between the subcritical Hopf bifurcation point and the saddle-node bifurcation point at coalescence of the lower stable node and the middle saddle. As [5-HT] increases, the CREB1 level becomes high via the saddle-node bifurcation. If thereafter [5-HT] decreases, the level of [CREB1] will be low via the subcritical Hopf bifurcation. However, as [piR-F] is large as 2*μ*M (blue), an irreversible switch leads to the sustained high level of [CREB1] in the system.  Figure 7(b) illustrates the range of [piR-F] on the right of the dash-dotted line that enables the system to transit from low to high under 5-HT stimulation (see shaded part in Figure 7(b)).

Thereby, the existence of irreversible and reversible switches, which decides whether the high [CREB1] level for long-term memory can be maintained or not, depends on chosen values of the parameters [miR-124] or [piR-F]. Thus, appropriate levels of miR-124 and piR-F are required for long-term memory formation.

**Figure 8:**
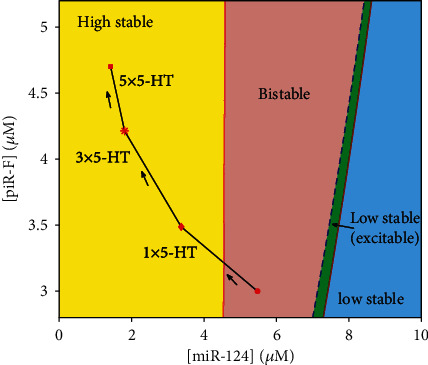
Bifurcation diagram in the ([miR‐124], [piR‐F]) parameter plane. The red solid line and blue dashed line depict the saddle-node bifurcation points (SN) and subcritical Hopf bifurcation points (subH), respectively.

To further investigate the synergistic mechanism of the epigenetic regulation through miR-124 and piR-F in the gene regulatory network, we consider [miR-124] and [piR-F] as parameters to perform a two-parameter bifurcation analysis in Figure 8. The ([miR‐124], [piR‐F]) parameter plane is divided into four regions by the saddle-node bifurcation and the Hopf bifurcation curves, that is, high stable region with one high-level stable steady state, low stable region with one low level stable steady state, bistable region with two stable steady states and a saddle, and excitable region with a low stable steady state, a high unstable steady state, and a saddle. As shown in Figure 8, ([miR‐124], [piR‐F) = (5.4749*μ*M, 3*μ*M) (red dot) locates in the bistable region, and the system stays in the low stable steady state before stimulation. One pulse, three pulses, and five pulses of 10*μ*M 5-HT cause the system to reach the high stable region with the value of ([miR‐124], [piR‐F]) as (3.3695*μ*M, 3.4877*μ*M) (red diamond), (1.8054*μ*M, 4.2139*μ*M) (red star), and (1.4192*μ*M, 4.7007*μ*M) (red square), respectively. However, only the accumulative effects of a stimulus protocol with five pulses of 5-HT can push the system to the attraction domain of the high-level steady state at [5‐HT] = 0*μ*M, but one or three pulses cannot. Therefore, with a starting point in the bistable region, the application of five pulses of 10*μ*M 5-HT is able to trigger the transition of the CREB1 concentration from a low- to a high-level steady state, and the high-level state persists even when 5-HT returns to 0*μ*M.

## 4. Discussion

In Aplysia, noncoding RNAs including miR-124 and piR-F as important regulators inhibit CREB1 mRNA and repress the promoter of CREB2, respectively, which reveal epigenetic mechanisms for regulating gene expression underlying long-term memory storage [7]. In this work, we develop a gene regulatory network model of two transcription factors (CREB1 and CREB2) regulated by two noncoding RNAs (miR-124 and piR-F). The two-parameter bifurcation diagram on the (([5‐HT], *V*_2_) parameter plane demonstrates diverse dynamical behaviors in the system and the existence of three codimension-2 bifurcation points including a cusp point, a Bogdanov-Takens bifurcation point, and a generalized Hopf bifurcation point. Besides that, varying the stimulus strength [5-HT] at different values of the negative feedback strength *V*_2_ generates various codimension-1 bifurcations such as saddle-node bifurcation, saddle-node invariant circle bifurcation, Hopf bifurcation, fold limit cycle bifurcation, and homoclinic bifurcation. Furthermore, the robustness analysis of system dynamics indicates that bistability and oscillations are robust to variations of all the parameters.

We validate the model by the numerical simulations on the experimental results in four stimulus protocols of one, three, four, and five short pulses of 5-HT. Simulation results illustrate that application of five pulses of 5-HT leads to a transient decrease in [miR-124] and increase in [piR-F] and induces sustained high level of [CREB1], which are in accordance with the experimental findings. Moreover, dynamical mechanisms of the epigenetic regulation in long-term memory formation are explored through bifurcation analyses considering [miR-124] and [piR-F] as parameters. When the system is bistable at basal level of 5-HT and monostable with a high steady state at stimulation level of 5-HT, the application of five pulses of 5-HT is able to trigger the transition of the CREB1 concentration from a low- to a high-level steady state, which persists high after stimulation. This confirms that five pulses of 5-HT can induce the sustained high level of CREB1 associated with long-term memory formation.

The bifurcation analyses of the model reveal divergent dynamics such as bistability and oscillation. We explicate the dynamical mechanisms underlying the epigenetic regulation in long-term memory formation through bistable switches in the model. Our results support the idea that the existence of irreversible and reversible switches, which decides whether the high [CREB1] level for long-term memory can be maintained or not, depends on the levels of [miR-124] or [piR-F] as controlling parameters. Therefore, bistability as a crucial dynamic property for long-term memory formation in *Aplysia* may be useful for further experimentation. In addition, noncoding RNAs may also induce oscillatory behaviors in other biological systems [31, 32]. The results of oscillatory dynamics produced from our model is expected to be helpful to the experimentalists for researches of other systems involving noncoding RNA regulation.

The simplified gene regulatory motif proposed in this study allows us to investigate dynamical mechanisms for epigenetic control of memory-related synaptic plasticity. However, experimental phenomena indicated that piR-F level exposure to 5-HT that leads to a time delay needs more time (about 3-4 hr) to be upregulated to a peak before dropping back to baseline [19]. Moreover, random fluctuations in gene expression exist universally in all kinds of organisms [33, 34], which have not been considered in this work. Thus, future studies will focus on time delay and stochastic dynamics in noncoding RNA-mediated regulation of synaptic plasticity.

## Figures and Tables

**Table 1:** Default parameter values of Equations (1)–(6).

Parameter	Value	Parameter	Value
[5‐HT] (*μ*M)	0	*g*_*i*_ (*μ*M min^−1^)	0.055
*λ* (*μ*M^−1^ min^−1^)	0.01	*δ* (*μ*M^−1^ min^−1^)	0.02
*g*_*p*_ (*μ*M min^−1^)	0.03	*γ* (min^−1^)	0.01
*d*_*i*_ (min^−1^)	0.01	*d*_*p*_ (min^−1^)	0.01
*g*_*m*1_ (*μ*M min^−1^)	0.0002	*g*_*m*2_ (*μ*M min^−1^)	0.001
*V*_1_ (*μ*M min^−1^)	2.5	*V*_2_ (*μ*M min^−1^)	1.5
*K*_1_ (*μ*M)	1	*K*_2_ (*μ*M)	0.45
*d*_*m*1_ (min^−1^)	0.005	*d*_*m*2_ (min^−1^)	0.005
*k*_*p*1_ (min^−1^)	0.1	*k*_*p*2_ (min^−1^)	0.1
*d*_*p*1_ (min^−1^)	0.01	*d*_*p*2_ (min^−1^)	0.01
*α* (*μ*M)	1	*β* (*μ*M)	2

## Data Availability

The data used to support the findings of this study are available from the corresponding author upon request.
